# Supplementary material for: Ectopic expression of pericentric HSATII RNA results in nuclear RNA accumulation, MeCP2 recruitment, and cell division defects
Source: Chromosoma. 2021 Feb 13;130(1):75–90. doi: 10.1007/s00412-021-00753-0 (PMC7889552; doi:10.1007/s00412-021-00753-0)
Supplement: Supplementary file 1 — (DOCX 3455 kb) [file 412_2021_753_MOESM1_ESM.docx]

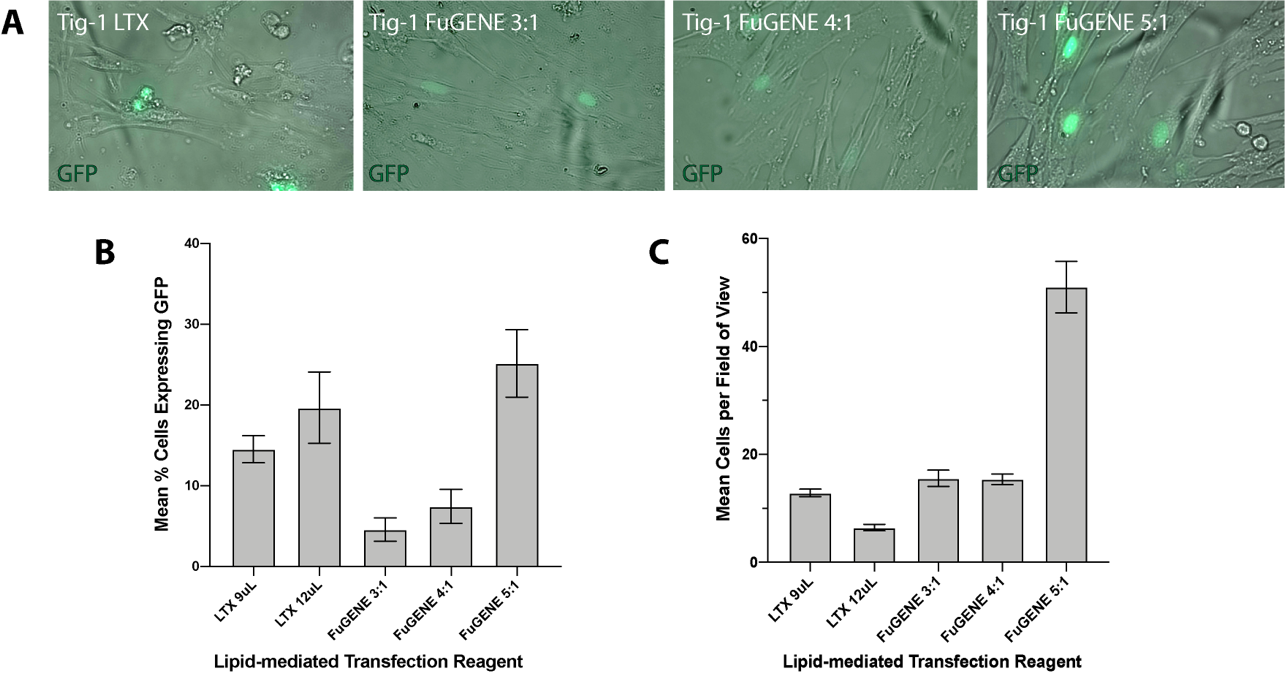


**Figure S1. Optimization of lipid-mediated transfection protocol for primary human fibroblasts. A)** Representative images of transient transfection of Tig-1 cells with GFP expression vector and (left to right): 9 uL of Lipofectamine LTX reagent, 3:1 ratio of FuGENE HD reagent to GFP vector, 4:1 ratio of FuGENE HD, and 5:1 ratio of FuGENE HD reagent to GFP vector. **B)** Transfection efficiencies using Lipofectamine LTX or FuGENE HD reagent in scaled concentrations. Mean percentage of cells with GFP expression out of total cells per field of view (± SEM). **C)** Cell viability of selected reagents and concentrations on transfected Tig-1 cells. Mean number of cells per field of view is shown (± SEM).


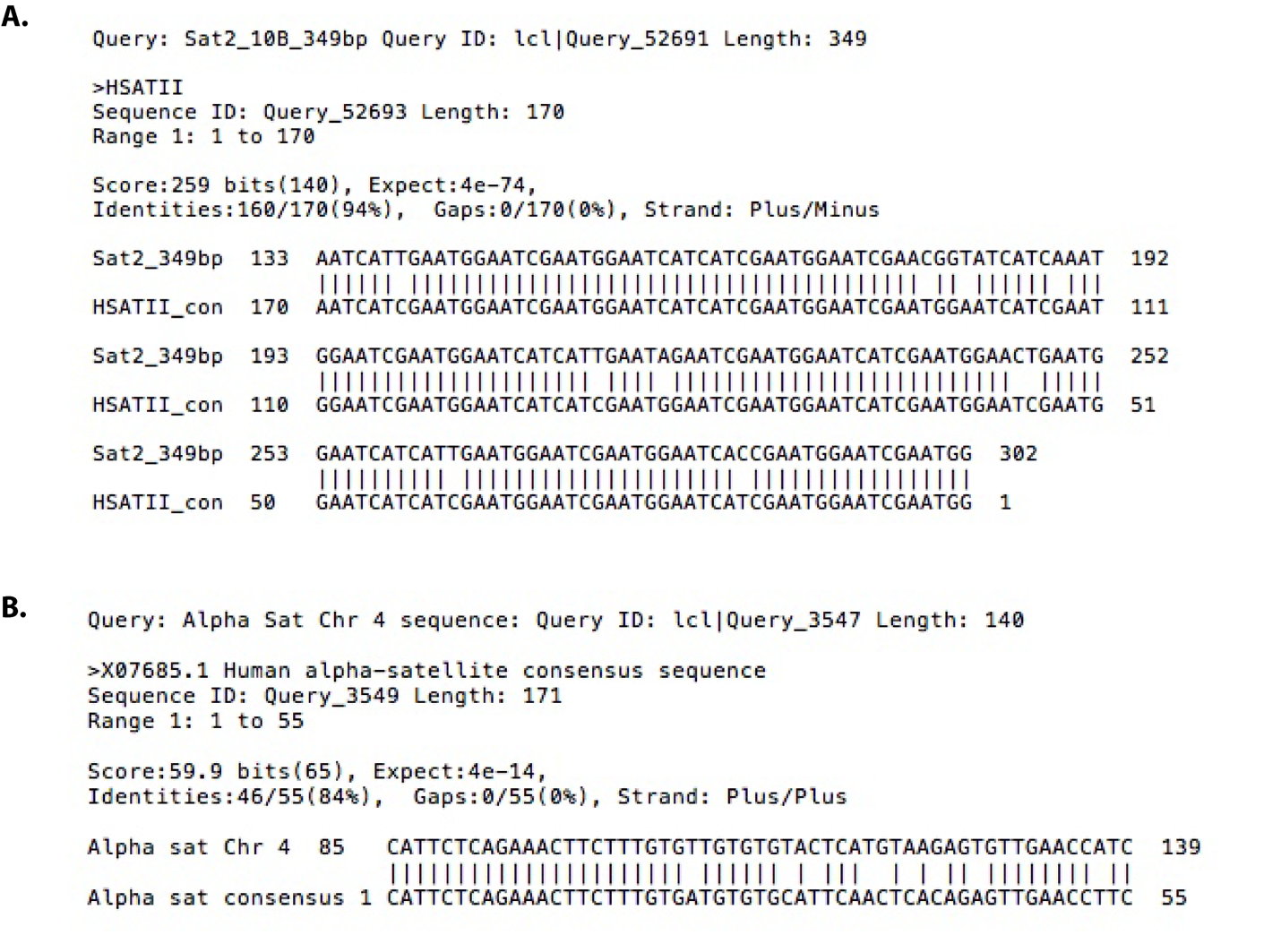


**Figure S2. Alignment of chromosome-specific expression clones to satellite consensus sequences. A)** Alignment of 349bp Chr7-specific HSATII variant to HSATII Repbase consensus sequence (HSATII_con). Alignment produced by Blast2seq. **B)** Alignment of 140bp Chr4-specific alpha satellite variant (Genbank M38467.1) to Repbase alpha satellite consensus sequence (Genbank X07685.1)


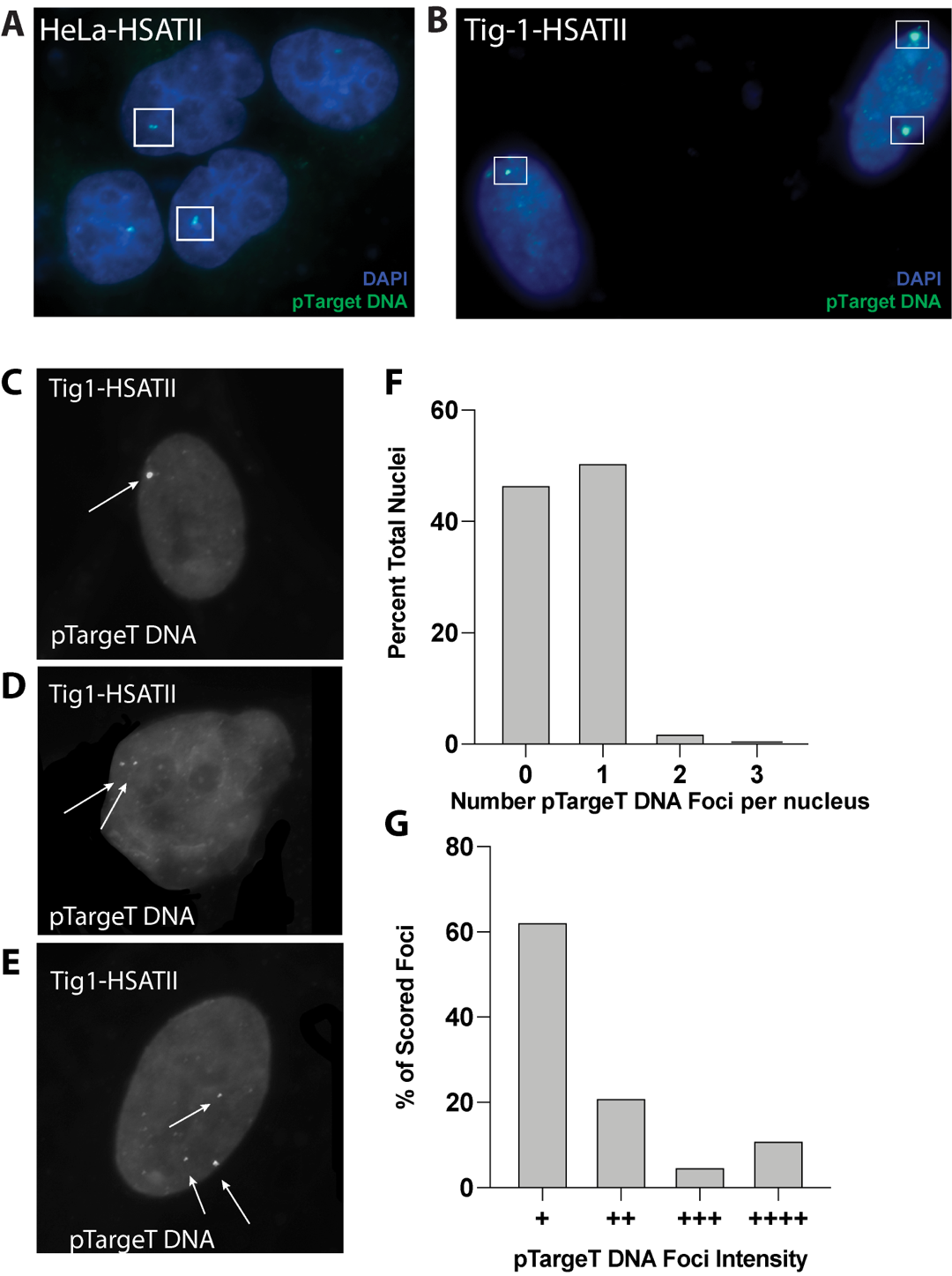


**Figure S3. pTargeT vector DNA integration at high efficiency in HSATII transfected Tig-1 cells.** **A-B)** Hybridization of pTarget (empty vector) shows integration into one or two genomic loci per nucleus in both HeLa **(A)** and Tig-1 **(B)** nuclei. FISH with labeled pTargeT backbone probe for Tig-1 showing **C)** Representative HSATII transfected nucleus with one site of integration. **D)** Representative HSATII transfected nucleus with two sites of integration**. E)** Representative HSATII transfected nucleus with three sites of integration. **F)** DNA FISH with the pTargeT backbone shows at least one site of pTargeT integration in 54.69% of HSATII transfected cells (N = 200). Due to incomplete DNA hybridization efficiency, not all nuclei displayed detectable pTargeT DNA signal. **G)** Relative intensity of scored foci was assigned to the following categories: + (dim), ++ (easily visible), +++ (very bright), or ++++ (extremely bright, saturated pixels). All foci were categorized and imaged using the same exposure time.


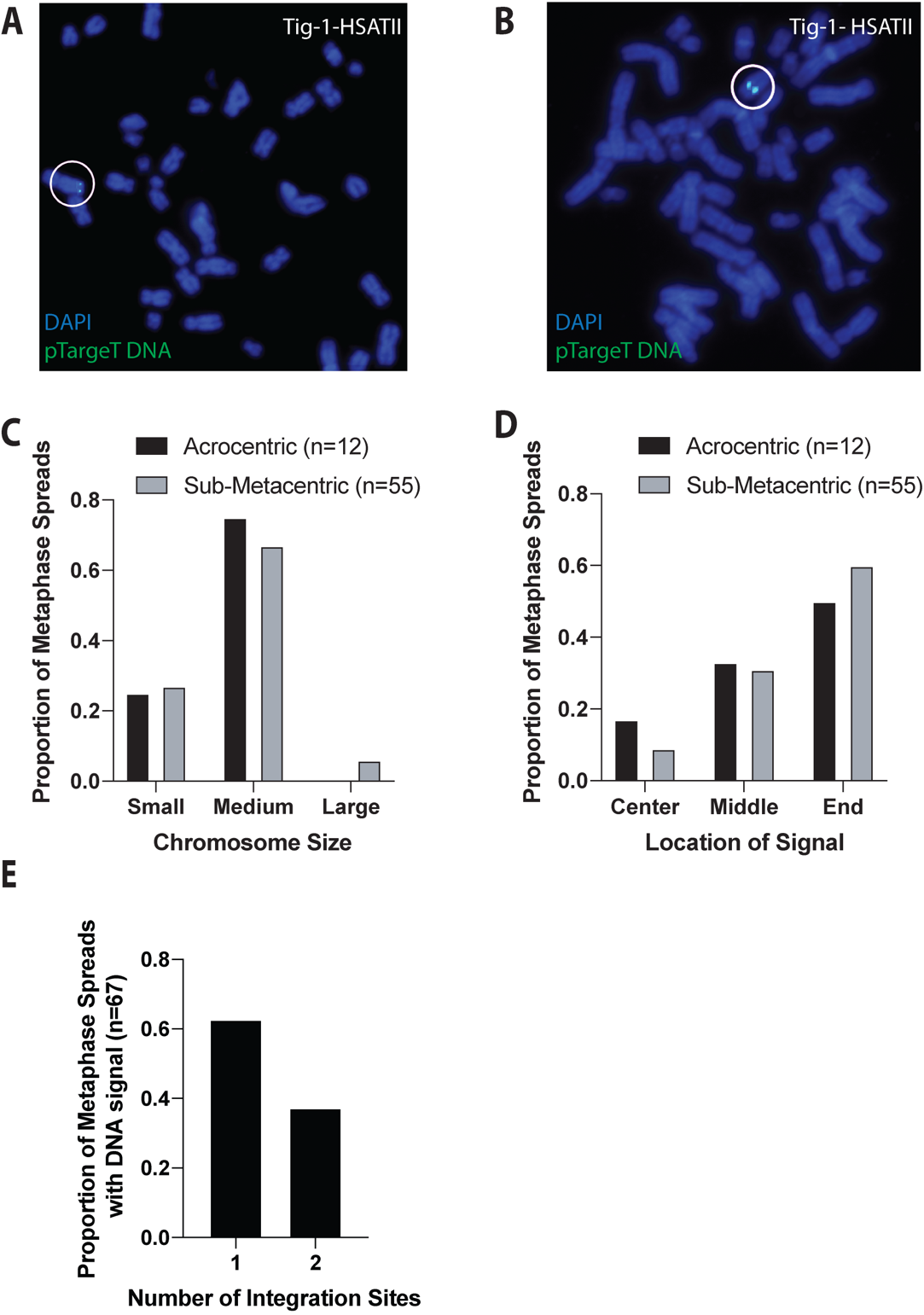


**Figure S4. Stable Tig-1 cell lines display random integration of pTargeT vector DNA into Tig-1 chromosomes. A-B)** Labeled pTargeT backbone probe detects integration sites of transfection vector containing HSATII DNA construct (green) in both **(A)** sub-metacentric and **(B)** acrocentric chromosomes on DAPI-stained metaphase spreads. Integration sites are indicated by circles and have been replicated on sister chromatids. **C-D)** Categorization of **(C)** chromosome size and **(D)** signal location were scored for both sub-metacentric and acrocentric chromosomes and demonstrate random integration on a variety of human chromosomes. Since most human chromosomes are categorized as “medium” size, the higher frequency of integration into medium-sized chromosomes likely reflects a random distribution of integration sites. **E)** The number of pTargeT vector DNA integration sites on HSATII transfected Tig-1 chromosome spreads was also scored. Data shown is for chromosome spreads with detectable pTargeT DNA hybridization signal.


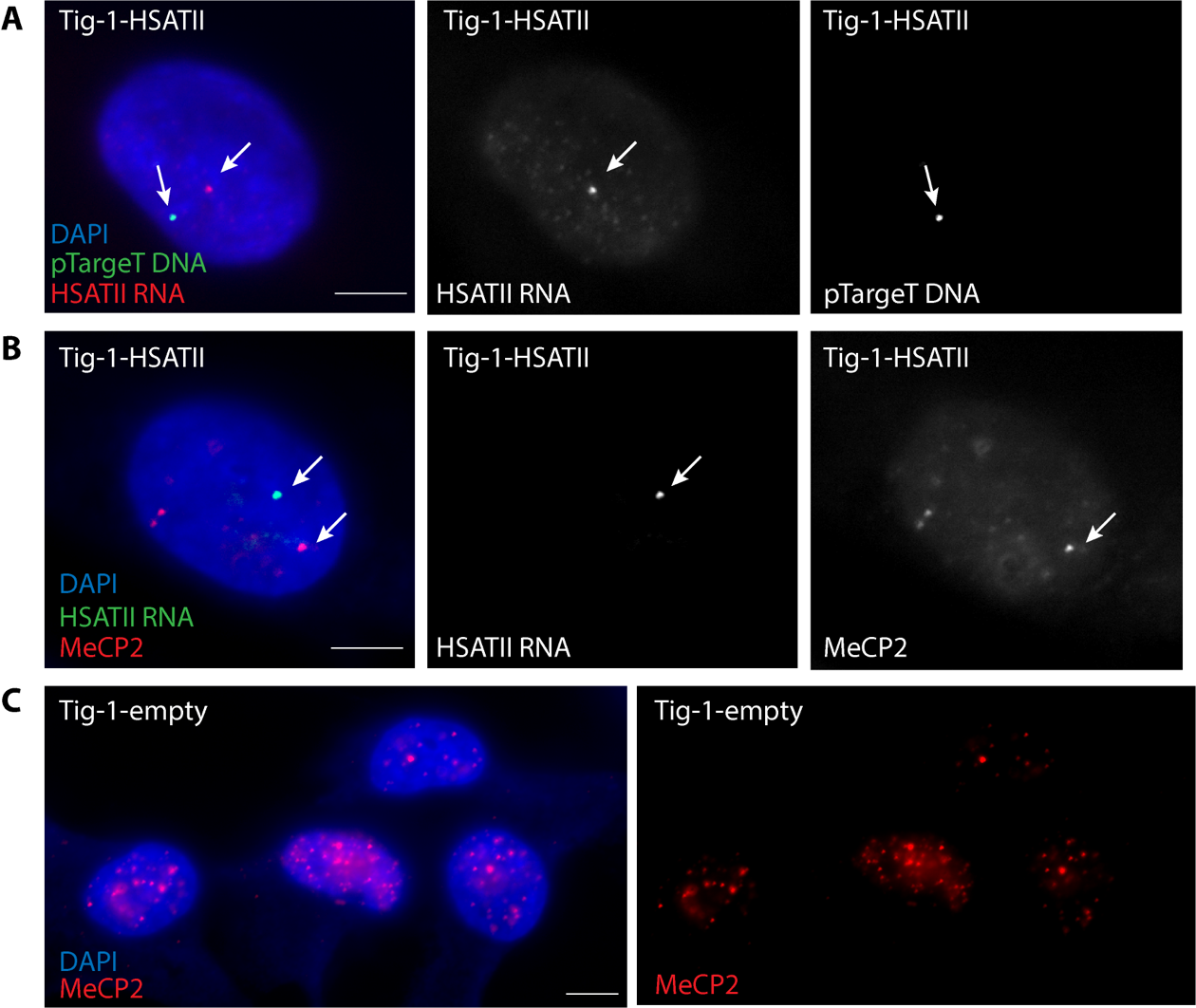


**Figure S5. Non-localized pattern of HSATII RNA with pTargeT DNA and MeCP2 in transfected Tig-1 cells. A)** In some Tig-1 cells transfected and expressing HSATII RNA, HSATII RNA (red) does not localize adjacent to sites of pTargeT vector integration (green). **B)** Tig-1 cells transfected and expressing HSATII (green) do not always recruit MeCP2 (red) to HSATII RNA accumulations (HSATII and MeCP2 distinct foci indicated by separate arrows). **C)** Tig-1 cells transfected with empty vector (pTargeT only) demonstrate MeCP2 nuclear foci (red) in a normal nuclear distribution. Scale bar, 5µm.


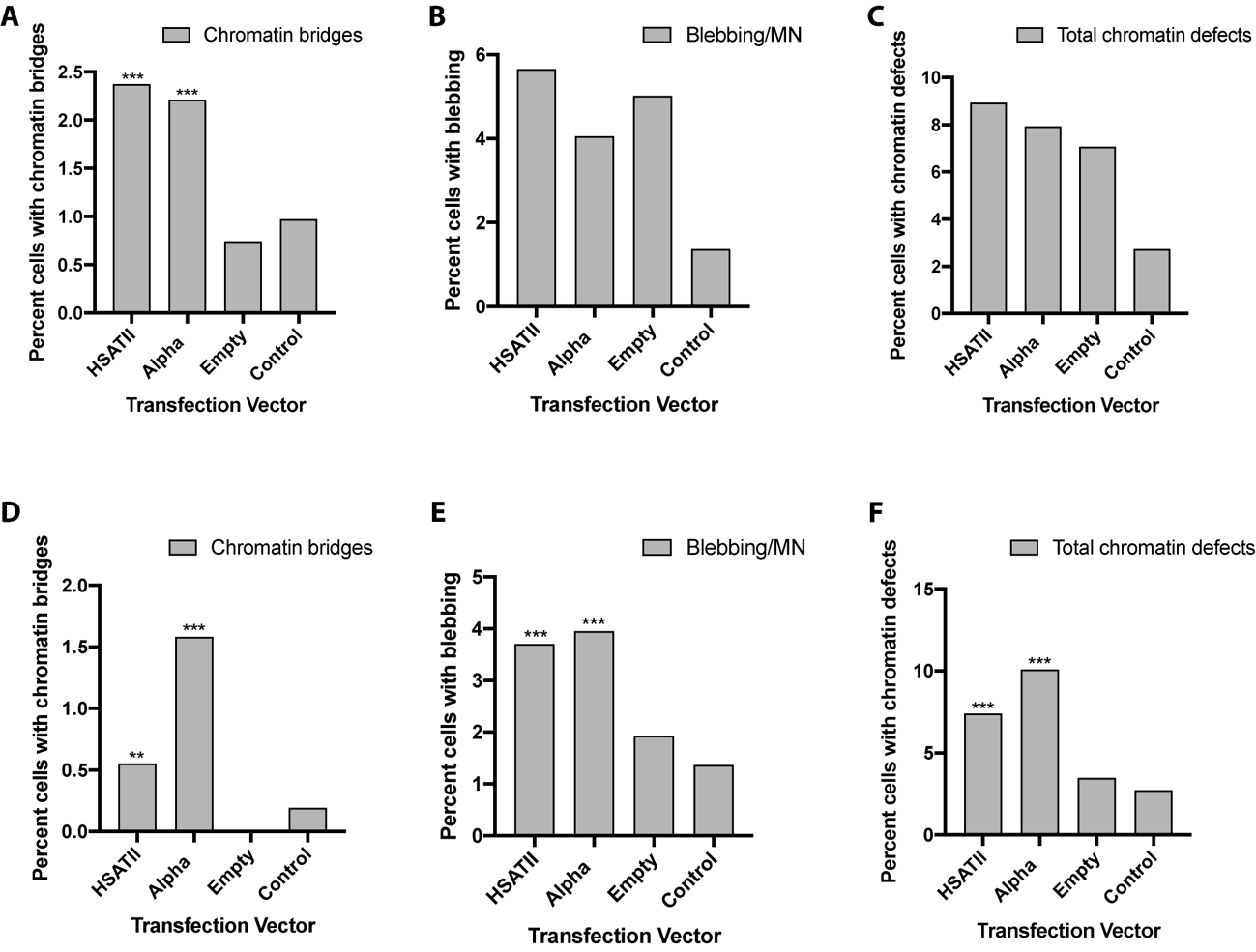


**Figure S6. Chromatin defect scoring from replicate HeLa (A-C) and Tig-1 (D-F) transfection experiments.** These scoring data reflect independent transfection experiments from those shown in Figure 4. Scoring of control HeLa or HeLa cells transfected with HSATII, a-sat or empty vector displaying percentage of cells with **A)** chromatin bridges, **B)** blebbing/MN and **C)** total chromatin defects out of 500 total cells for each transfection vector used. Percentages of control Tig-1 or Tig-1 cells transfected with HSATII, a-sat or empty vector displaying percentage of cells with **D**) chromatin bridges**,** **E**) blebbing/MN and **F)** total chromatin defects from 500 total cells per transfection vector. Data in this figure are from independent transfections from those shown in Fig. 4, but scoring and chi-square statistical analysis were performed in an identical manner.


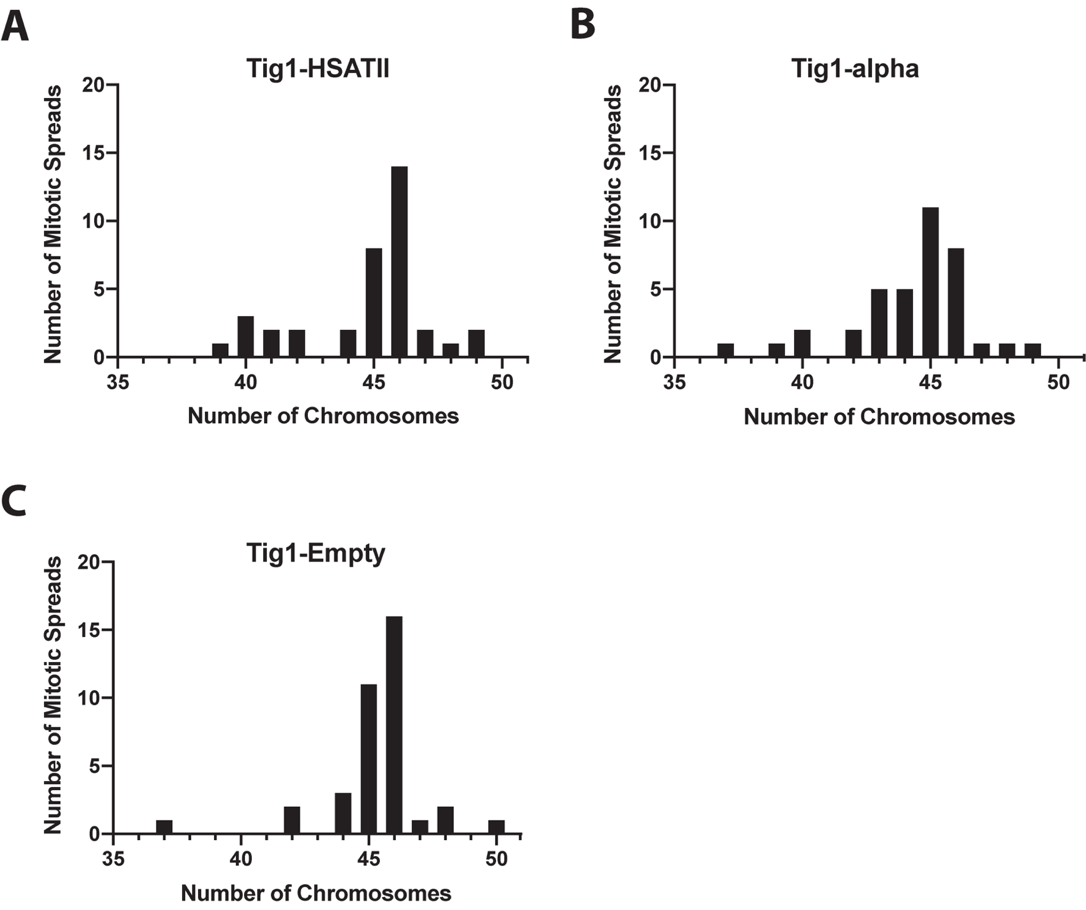


**Figure S7. Karyotype analysis of Tig-1 transfected cells.** Tig-1 cells were arrested and harvested for mitotic spread analysis 60 days following transfection. **A)** Number of chromosomes scored in each mitotic spread in HSATII transfected cells (n= 37). **B)** Number of chromosomes scored in each mitotic spread in alpha-sat transfected cells. **C)** Number of chromosomes scored in each mitotic spread in empty-vector transfected cells.
